# Impact of the Improved Publicly-funded Newborn Hearing Screening Program

**DOI:** 10.31662/jmaj.2024-0344

**Published:** 2025-03-28

**Authors:** Nao Doi, Ichiro Fukunaga, Taisuke Kobayashi, Kahori Hirose, Masamitsu Hyodo, Masanori Teshima

**Affiliations:** 1 Department of Rehabilitation, Kochi Medical School Hospital, Kochi, Japan; 2 Department of Otolaryngology, Kochi Medical School, Kochi, Japan; 3 Susaki Public Health and Welfare Office, Kochi Prefectural Government, Kochi, Japan; 4 Rehabilitation and Welfare Center, Kochi Prefectural Government, Kochi, Japan; 5 Department of Otolaryngology, Takanoko Hospital, Ehime, Japan; 6 Department of Otolaryngology, Hosogi Hospital, Kochi, Japan

**Keywords:** newborn hearing screening, public funding, congenital hearing loss, early intervention

## Abstract

**Introduction:**

To increase the coverage rate and effectiveness, universal newborn hearing screening (NHS) should be financed by public funding rather than individuals. This study investigated the impact of the publicly-funded NHS program on the detection rate and the time to diagnosis and initiation of intervention for children with congenital hearing loss.

**Methods:**

We compared two groups: one group included newborns born between April 2011 and March 2016 who either did not pass NHS or were referred due to high risk (Group 1); the other group included newborns born between April 2017 and March 2022 who met the same criteria (Group 2). The screening costs of Group 1 were covered by the guardians’ payments, whereas those of Group 2 were covered by public funding. The NHS program in Group 2 exhibited improved screening methods, course, and timing of diagnostic hearing tests for referred newborns. The number of detected newborns with hearing impairment, the period between birth and the initial visit to a diagnostic institution, and the time to intervention were evaluated.

**Results:**

Group 2 had more newborns with hearing loss (n = 51) than Group 1 (n = 32), representing a significant difference (p = 0.005). Group 2 had more children with bilateral hearing loss (n = 29) than Group 1 (n = 21), but the difference was not significant. The duration until the diagnostic test was significantly reduced in Group 2 (58 days in Group 1 vs. 35 days in Group 2). The duration of intervention also was significantly reduced in Group 2 (147 days vs. 99 days).

**Conclusions:**

The improved program based on public funding achieved an increased number of detected infants with hearing loss. Additionally, it shortened the durations until the first diagnostic test to an institution and intervention. The new NHS program funded by local governments achieved improved effectiveness by unifying the screening method, the course of diagnostic hearing examination, and the follow-up.

## Introduction

Universal newborn hearing screening (NHS) has been introduced worldwide for the early detection of hearing loss in newborns in the last two decades, and many studies have reported its effectiveness ^(1)^. It has been demonstrated that early detection and early intervention significantly influence the acquisition of language development in children with hearing impairment ^(2), (3), (4)^. Although NHS requires a high coverage rate, the actual screening rate in Japan was approximately 90% in 2019 ^(5)^. The absence of an increase in the coverage rate was thought to be a consequence of the lack of financial support in Japan ^(6)^. Recent policies have focused on increasing the NHS coverage rates through public expense rather than through self-pay. However, there are no reports regarding the effect of public expense on the detection rate and early intervention in children with hearing impairment.

Direct comparisons of programs across countries are difficult because NHS programs depend on the economic status and medical insurance system of each country ^(7), (8), (9)^. In many countries or regions, NHS is covered by health insurance or public expense; in others, the NHS cost is paid privately. In Japan, NHS is not covered by public health insurance. The NHS model project was initiated in Japan in 2001; it has been operated with modifications by each prefecture since 2007. According to a survey by the Ministry of Health, Labour, and Welfare, only 52.6% of local governments provided public funding in 2019 ^(5)^. Until 2015, only newborns whose parents paid for the costs were able to receive NHS in Kochi Prefecture, Japan. In 2016, the NHS program in Kochi Prefecture was improved. Since April 2017, NHS has been covered by public expense in all municipalities, extending the program to all newborns without paying screening costs. The new program established the screening methods, as well as the course and timing of diagnostic hearing tests for referred newborns; The new program made it possible to oblige maternity hospitals to implement NHS using not automated oto-acoustic emissions (aOAE) but automated auditory brain response (aABR). High-risk newborns also underwent NHS as other newborns without risks. It also made it possible to obtain the consent of guardians to report the results of NHS to the government. Therefore, public health nurses can follow up on the referred newborns. The purpose of this study is to explore the effects of this improved NHS program, supported by public funding, on the detection rate, duration until diagnosis, and time of intervention among children with congenital hearing loss.

## Materials and Methods

**Figure 1.**
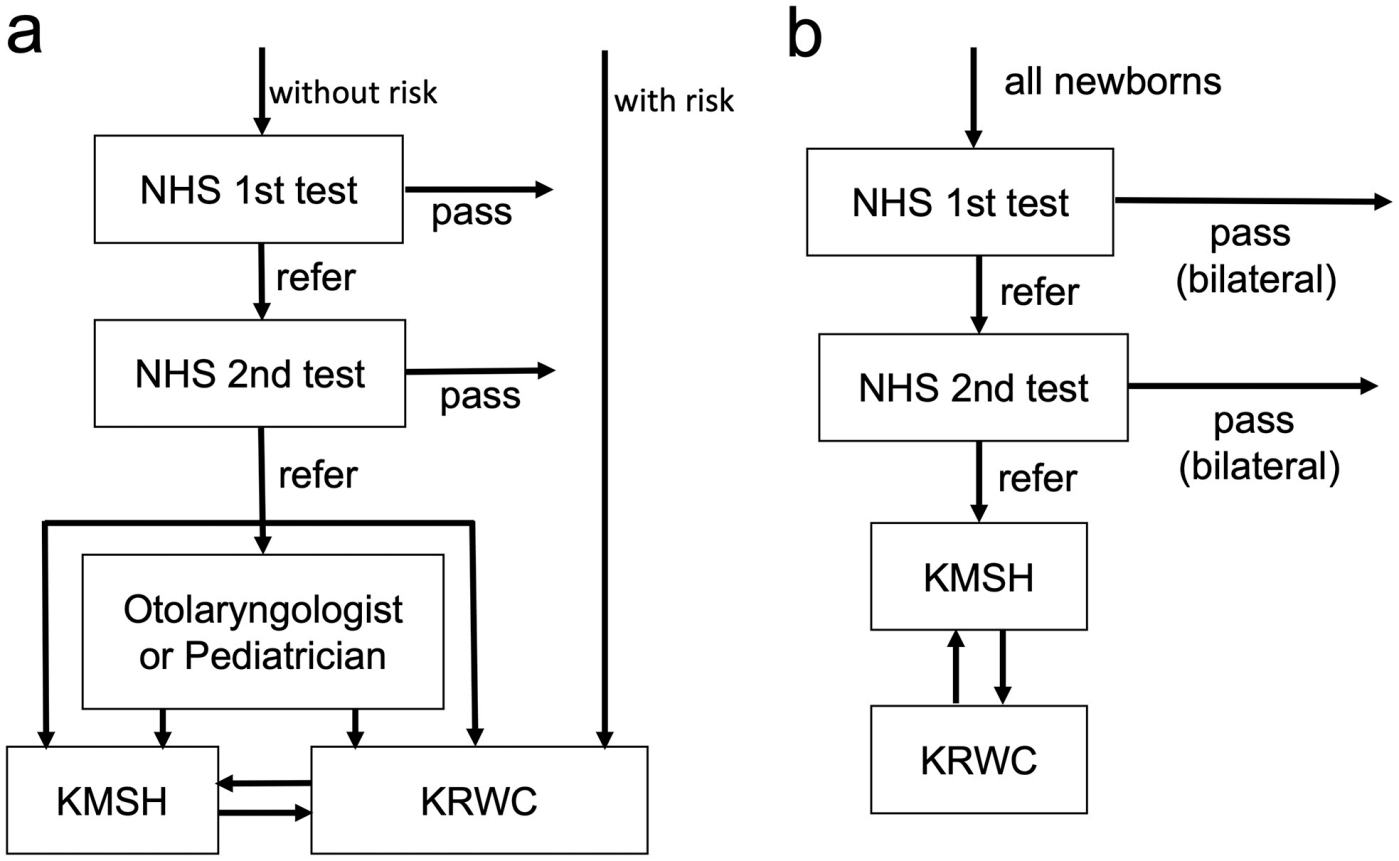
NHS flowchart. a: NHS program in Group 1 (April 2011 to March 2016), b: NHS program in Group 2 (April 2017 to March 2022). NHS: Newborn Hearing Screening, KMSH: Kochi Medical School Hospital, KRWC: Kochi Prefectural Rehabilitation and Welfare Center.

Two institutions in Kochi Prefecture provide examinations for pediatric hearing loss: Kochi Medical School Hospital (KMSH) and Kochi Prefectural Rehabilitation and Welfare Center (KRWC). Until 2016, diagnostic auditory assessment in NHS and hearing testing for high-risk infants were performed at these two institutions (Figure 1a). Since April 2017, the hearing assessments have been carried out in KMSH, with referral to KRWC only if hearing aids are required (Figure 1b). Therefore, it is possible to investigate all cases of congenital deafness found in Kochi Prefecture by analyzing patients in both institutions.

This study included newborns and infants who visited one of the two institutions for diagnostic auditory assessments. The patients were divided into two groups for comparison: children who did not pass NHS or were referred due to hearing loss or high risk, born between April 2011 and March 2016 (Group 1); and children who did not pass NHS or were referred due to high risk, born between April 2017 and March 2022 (Group 2). In the former group, screening was performed if guardians agreed to pay the screening cost of 5,000-10,000 JPY. In the latter group, screening was paid for by the municipalities in Kochi Prefecture. Children with hearing loss due to exudative otitis media, children who moved out of Kochi Prefecture after NHS, and children who died were excluded.

The programs differed between groups; the flowcharts are shown in Figure 1a and 1b. In Group 1, the unified program was not defined; aABR or aOAE was used for screening, depending on the hospital. Neonates who did not pass were referred to the Department of Otolaryngology in KMSH or KRWC for diagnostic hearing assessment. However, some children were followed up at the pediatrics department, referred to an otolaryngology department outside of these two facilities, or referred to KMSH or KRWC after diagnostic ABR had been performed in their own hospital (Figure 1a). In Group 2, all cases were screened using aABR, in accordance with the program defined by Kochi Prefecture; if they did not pass binaural testing even in the second screening assessment, all cases were referred to the Department of Otolaryngology in KMSH (Figure 1b). After an examination, such as otoscopy, distortion product OAE and diagnostic ABR were performed, along with auditory steady-state response (ASSR) if required. Subsequently, patients with moderate or greater bilateral hearing loss were referred to KRWC, where behavioral observation audiometry (BOA) was performed, and hearing aids were provided. Cases with mild or unilateral hearing loss underwent regular hearing checks at KMSH or KRWC.

Because the programs differed between the two groups, the following parameters were defined as the study endpoints.

### ①Prevalence of children with hearing impairment

The frequencies of bilateral and unilateral hearing loss were calculated as a proportion of the total number of births. The number of births for each year was assessed using monthly reports from Kochi Prefecture ^(10)^.

### ②Duration until first visit

Number of days between birthday and first visit to one of the two institutions.

### ③Intervention date

Defined as; 1) the day when any other auditory assessments (ASSR or BOA) were performed for the first time after diagnostic ABR for children referred to KMSH, and 2) the day of the first visit to KRWC for children referred to KRWC.

Statistical analyses were conducted using IBM SPSS Statistics Version 26 (IBM Japan, Tokyo, Japan). Since most patients in this retrospective study are not currently seen at our clinic, they could declare that they would not participate in the study by opting out instead of obtaining written informed consent. The study protocol was approved by the Ethics Committee of Kochi Medical School and the Treatment and Welfare Center in Kochi.

## Results

Table 1 shows the characteristics of both groups and their corresponding results. In Kochi Prefecture, 25,852 babies were born between April 2011 and March 2016; 21,631 babies were born between April 2017 and March 2022. Group 1 comprised 65 infants (41 boys and 24 girls), of which 51 were first evaluated at KMSH and 14 were first evaluated at KRWC. Group 2 comprised 148 infants (91 boys and 57 girls), of which 147 were referred to KMSH; only one was referred to KRWC without NHS. Eight children in Group 1 and 11 children in Group 2 dropped out before the final diagnosis. In Group 1, 38 (58.5%) were detected by the NHS, whereas in Group 2, 147 (99.3%) were detected. Groups 1 and 2 included 22 (33.8%) and 53 (35.8%) high-risk newborns, respectively; there was no significant difference between groups.

**Table 1.** Characteristics of Each Group and Results.

	Group 1	Group 2	
	Nr. of newborn: 25,852	Nr. of newborn: 21,631	
n (rate to newborns)	65 (0.25%)	148 (0.68%)	*p*=0.000^a^
Sex			
boy	41	91	NS^a^
girl	24	57	NS^a^
Hearing loss	32 (0.12%)	51 (0.24%)	*p*=0.005^a^
unilateral	11 (0.04%)	22 (0.10%)	*p*=0.015^a^
bilateral	21 (0.08%)	29 (0.13%)	*p*=0.077^a^
Until first visit (median, days)	58.0	35.0	*p*=0.000^b^
Until intervention (median, days)	147.0*	99.0**	*p*=0.037^b^

*: median of 29 children, **: median of 48 children a: χ2 test, b: Mann-Whitney’s U test

### Prevalence of children with hearing impairment

Thirty-two children in Group 1 (42.9%) were finally diagnosed with unilateral or bilateral hearing loss, constituting 0.12% of the newborns. Among them, 11 (0.04% of all newborns) had unilateral hearing loss; 21 (0.08% of all newborns) had bilateral hearing loss. Group 2 included 51 infants with hearing loss (34.4% of Group 2; 0.24% of all newborns). Of these, 22 (0.10% of all newborns) had unilateral hearing loss and 29 (0.13% of all newborns) had bilateral hearing loss. Thus, there were significantly more newborns with hearing loss in Group 2 (χ^2^ test, p = 0.005). Group 2 had more children with bilateral hearing loss than Group 1, but the difference was not statistically significant.

### Duration until first visit

**Figure 2.**
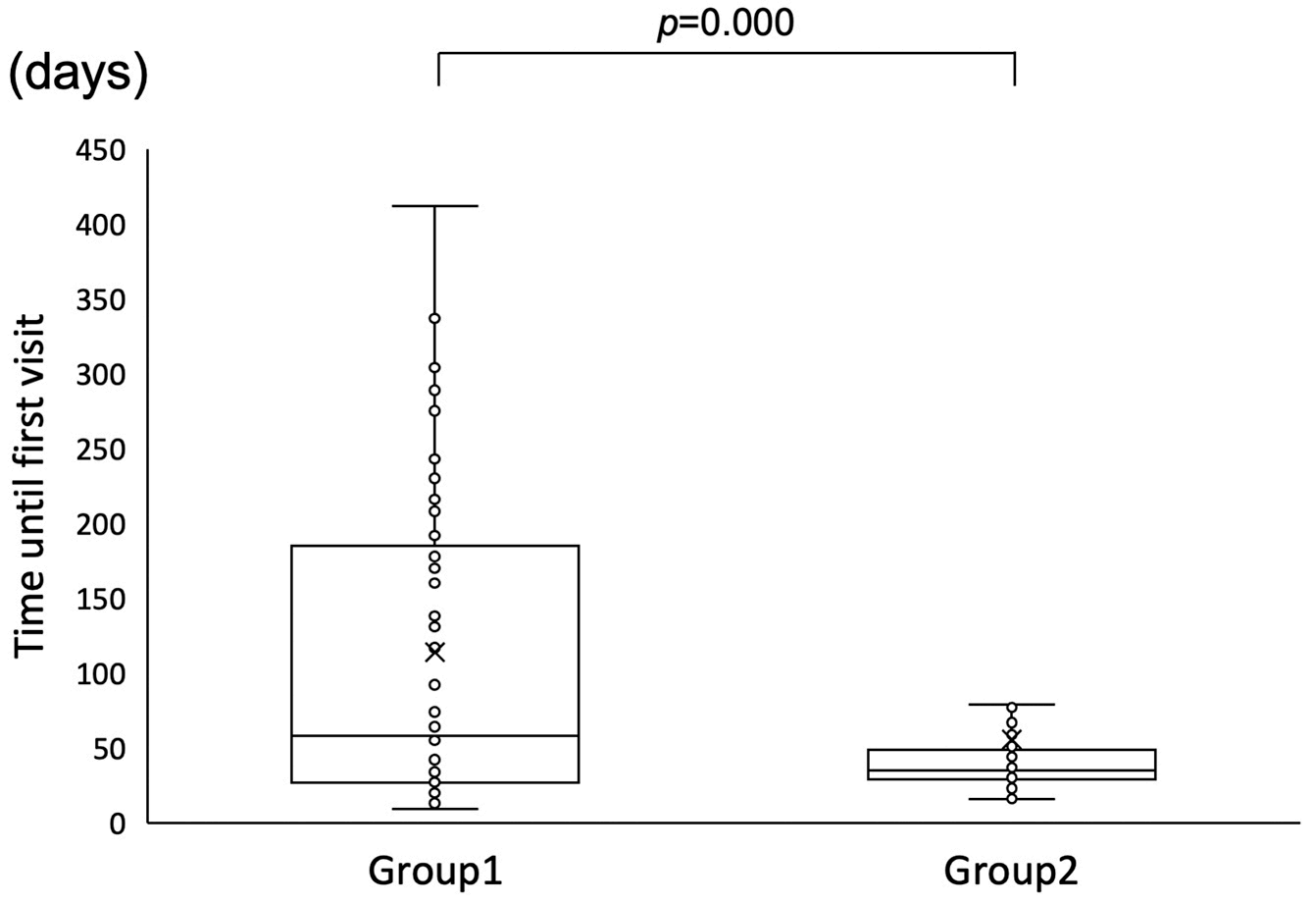
Duration until the first visit to a diagnostic institution. X indicates the average of each group.

The median durations until the first visit were 58.0 days in Group 1 and 35.0 days in Group 2 indicating a significant difference (Mann-Whitney U test, p = 0.000) (Figure 2).

### Duration until intervention

**Figure 3.**
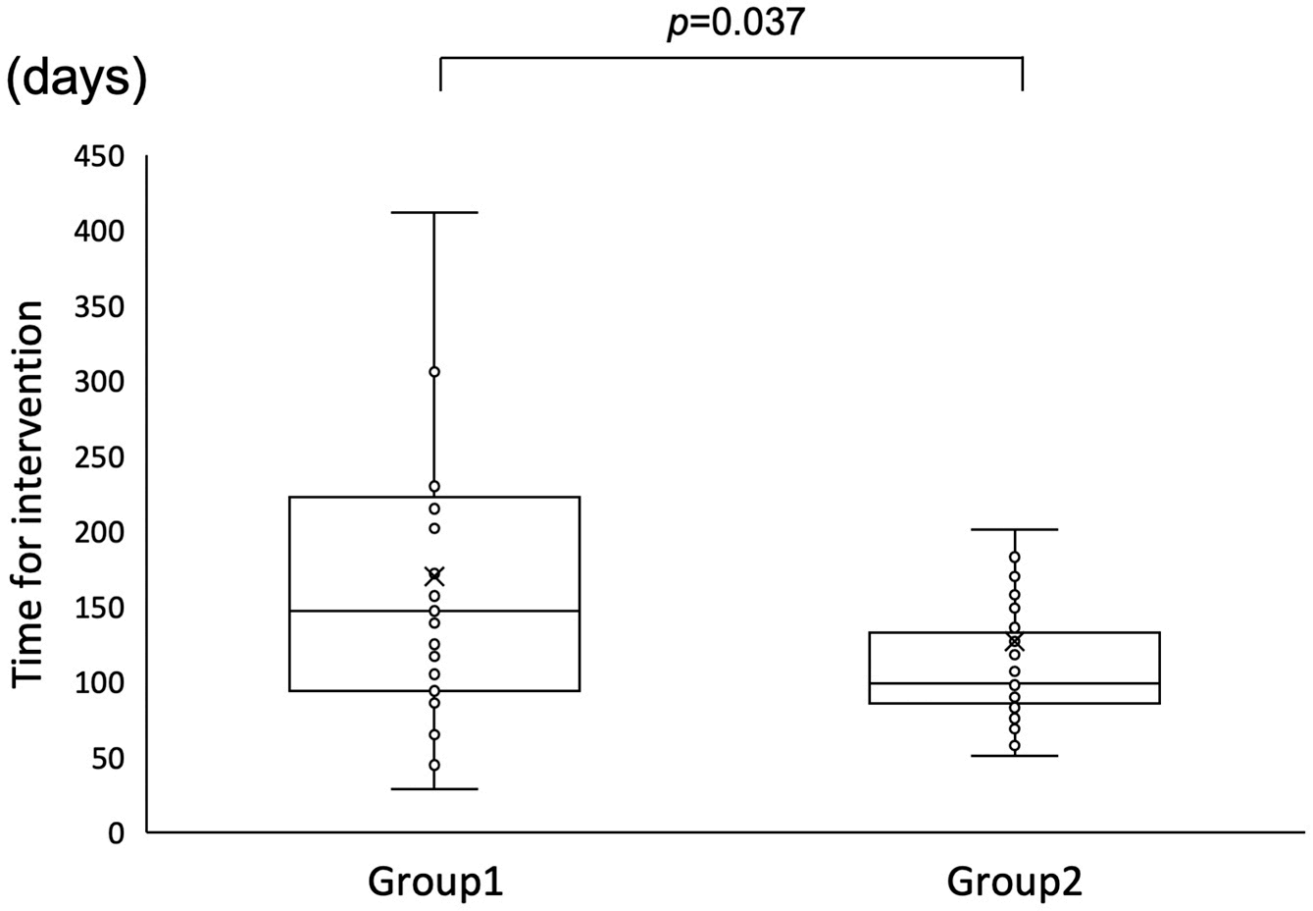
Duration until intervention. X indicates the average of each group.

The intervention date was unknown for three of the 32 children with hearing loss in Group 1; among the remaining 29 patients, the median age at the time of intervention was 147.0 days. In Group 2, two of the 51 children with hearing loss dropped out, and the intervention date for one was unknown. Among the remaining 48 patients, the median age at the time of intervention was 99.0 days. The duration until intervention was significantly shorter in Group 2 (Mann-Whitney U test, p = 0.037) (Figure 3).

## Discussion

Early detection and intervention in children with congenital hearing loss are crucial for language development ^(2), (3), (4)^. This requires an increase in the NHS coverage rate. A position paper from the United States recommended a minimum 95% coverage rate for NHS and a 95% follow-up rate ^(11)^. In 2019, the NHS coverage rate was 90.8% in Japan ^(5)^. To increase the NHS coverage rate, the Japanese government has introduced policies for the adoption of publicly-funded NHS. Previously, only 52.6% of municipalities were implementing public expense, including those with partial public expense ^(5)^. In Kochi Prefecture, until 2015, all NHS costs were paid by guardians. However, since 2017, the NHS program has improved, and all costs are paid by the municipality. Consequently, the coverage rate reached an average of 99.2% between 2017 and 2020 ^(12)^, an increase relative to 74.3% in 2013 (unpublished data from a questionnaire survey for each obstetric department in Kochi Prefecture). The present study investigated the possibility that the aforementioned changes might increase the number of detected children with hearing loss.

In this study, the number of detected newborns with hearing loss was 32 in Group 1 and 51 in Group 2. The prevalence of children diagnosed with hearing loss significantly increased from 0.12% to 0.24%. The prevalence of children with unilateral hearing loss was significantly higher in Group 2 than in Group 1. The prevalence of bilateral hearing loss was 0.08% in Group 1 and 0.13% in Group 2, but the difference was not statistically significant. In a report from Japan using an NHS program similar to that of Group 2, infants with bilateral hearing loss were detected in 0.14% of newborns ^(6)^, consistent with the results of the present study. Our results showed that the improved program could achieve a higher detection rate for newborns with hearing loss; however, the detection rate for bilateral hearing loss did not significantly change. Considering the prevalence of congenital hearing loss, there is a need to validate these findings in a larger population. Further studies are required to assess the effect of public expense on the detection rate of infants with hearing impairment.

There were several reasons for the increased detection rate of children with hearing loss in Group 2. The most prominent factor is the increase in the NHS coverage rate, which I mentioned above. This is largely because the financial burden on parents has been reduced through public funding. Another reason is that midwives now always explain to parents the necessity and usefulness of the NHS. Hsu et al. ^(13)^ reported that the coverage rate in Taipei increased from 87.34% to 99.64% after the cost was paid by Taipei City. Scheepers et al. ^(14)^ reported that the most common reason for NHS refusal in South Africa was the cost (72%). The next most common reason was a lack of knowledge regarding NHS among parents (62%) ^(14)^. Reduced financial burden and information about the usefulness of the NHS would be major factors in increasing the coverage rate of the NHS.

Several other factors may have contributed to the increase in the detection rate of children with hearing loss. The second factor is that almost all high-risk newborns in Group 2 are receiving NHS. In Group 1, high-risk newborns directly underwent diagnostic ABR at the discretion of the pediatricians, however, some high-risk newborns did not undergo sufficient hearing tests. In Group 2, all but one of the high-risk children were screened after their general condition had stabilized or when they had reached the equivalent of 34 weeks. Before the widespread adoption of NHS, most high-risk children were directly subjected to diagnostic ABR testing. However, NHS recently has gained greater acceptance among high-risk children ^(15), (16), (17)^, as demonstrated in the present study.

Thirdly, all cases were screened using only aABR in Group 2. Group 1 used aOAE for screening at a few hospitals. Initial screening using aOAE combined with aABR is considered more economical ^(18), (19)^. In the NHS with aOAE, however, children with auditory neuropathy may show false-negative ^(20)^, so they might be missed in Group 1.

Fourthly, “refer” cases were followed up by public health nurses in Group 2, which may have reduced drop-out. In the improved program (Group 2), the results of NHS and diagnostic tests were reported to the municipality, and follow-up was carried out by public health nurses. This approach offers the advantage of quality control by the prefectural government. In recent years, the importance of a tracking system for referred babies has been addressed ^(21)^. Park et al. ^(22)^ reported that governmental support for both timely interventions and the introduction of a web-tracking system was mandatory to ensure a successful NHS program. We also support the notion that a tracking system should be introduced in NHS programs.

Considering the social cost of NHS, several studies have reported simulations concerning the effects of NHS on healthcare, rehabilitation, society, and the economy. Sharma et al. ^(23)^ reported the first evaluation to assess the cost-effectiveness of NHS from the Australian healthcare perspective. They compared NHS and targeted screening (diagnostic ABR for high-risk children) using real-world data, demonstrating the cost-effectiveness of NHS. Because Australia is a leading country in the treatment of hearing loss, it seems reasonable that such a cost-effective model could be adopted by other countries. Therefore, public NHS funding is considered appropriate from a policy perspective.

In this study, the duration until the initial diagnostic hearing assessment was significantly reduced from 58 days to 35 days with the improved program. The duration until intervention also was reduced from 147 days to 99 days. The main reason for these significant reductions in the durations until diagnostic hearing assessment and intervention is probably due to the strict definition of a diagnostic hearing procedure for the referred child in the improved NHS program. Another factor may have been the follow-up of children referred by public health nurses. These factors may be related to the fact that the availability of public funding has enabled local governments to track referred children and provide interventions.

There was a limitation in the present study. This was a historical retrospective study, and Groups 1 and 2 differed in their programs, so it was not possible to directly evaluate only the effect of public funding. However, as previously mentioned, public funding has allowed the government to become more involved in NHS programs, which has increased the overall value of these programs.

### Conclusions

The improved publicly-funded NHS program achieved an increased number of detected newborns with hearing loss. Additionally, it shortened the duration until the first diagnostic visit to an institution and intervention. The new NHS program funded by local governments achieved improved effectiveness by unifying the screening method, the course of diagnostic hearing examination, and the follow-up. These results highlight the effectiveness of publicly-funded NHS.

## Article Information

### Conflicts of Interest

None

### Acknowledgement

The authors would like to thank Dr. Kiyofumi Gyo for useful discussions. We also thank the speech therapists of the Kochi Prefectural Rehabilitation and Welfare Center. We express our deepest gratitude to the staff of Kochi Prefectural Government for their greatest effort in introducing the new NHS program.

### Author Contributions

Nao Doi: Investigation, Formal analysis, Writing-original draft, Ichiro Fukunaga: Conceptualization, Methodology, Taisuke Kobayashi: Methodology, Writing-Reviewing and Editing, Project administration, Kahori Hirose: Investigation, Data curation, Masamitsu Hyodo: Supervision, Naonori Teshima: Supervision.

### Approval by Institutional Review Board (IRB)

Approval was received from the Ethics Committee of the Kochi Medical School (Number 30-11); and Treatment and Welfare Center, an affiliated facility of Kochi Prefecture (gan-ko-ryoiku-1034, October 9, 2019).
